# Transcatheter heart valve interventions for patients with rheumatic heart disease

**DOI:** 10.3389/fcvm.2023.1234165

**Published:** 2023-09-13

**Authors:** Hellmuth Weich, Philip Herbst, Francis Smit, Anton Doubell

**Affiliations:** ^1^ Division of Cardiology, Department of Medicine, Faculty of Health Sciences, Stellenbosch University, Cape Town, South Africa; ^2^ Robert W.M. Frater Cardiovascular Research Centre, University of the Free State, Bloemfontein, South Africa

**Keywords:** rheumatic heart disease, transcatheter intervention, mitral, aortic stenosis, regurgitation

## Abstract

Rheumatic heart disease [RHD] is the most prevalent cause of valvular heart disease in the world, outstripping degenerative aortic stenosis numbers fourfold. Despite this, global resources are firmly aimed at improving the management of degenerative disease. Reasons remain complex and include lack of resources, expertise, and overall access to valve interventions in developing nations, where RHD is most prevalent. Is it time to consider less invasive alternatives to conventional valve surgery? Several anatomical and pathological differences exist between degenerative and rheumatic valves, including percutaneous valve landing zones. These are poorly documented and may require dedicated solutions when considering percutaneous intervention. Percutaneous balloon mitral valvuloplasty (PBMV) is the treatment of choice for severe mitral stenosis (MS) but is reserved for patients with suitable valve anatomy without significant mitral regurgitation (MR), the commonest lesion in RHD. Valvuloplasty also rarely offers a durable solution for patients with rheumatic aortic stenosis (AS) or aortic regurgitation (AR). MR and AR pose unique challenges to successful transcatheter valve implantation as landing zone calcification, so central in docking transcatheter aortic valves in degenerative AS, is often lacking. Surgery in young RHD patients requires mechanical prostheses for durability but morbidity and mortality from both thrombotic complications and bleeding on Warfarin remains excessively high. Also, redo surgery rates are high for progression of aortic valve disease in patients with prior mitral valve replacement (MVR). Transcatheter treatments may offer a solution to anticoagulation problems and address reoperation in patients with prior MVR or failing ventricles, but would have to be tailored to the rheumatic environment. The high prevalence of MR and AR, lack of calcification and other unique anatomical challenges remain. Improvements in tissue durability, the development of novel synthetic valve leaflet materials, dedicated delivery systems and docking stations or anchoring systems to securely land the transcatheter devices, would all require attention. We review the epidemiology of RHD and discuss anatomical differences between rheumatic valves and other pathologies with a view to transcatheter solutions. The shortcomings of current RHD management, including current transcatheter treatments, will be discussed and finally we look at future developments in the field.

## Introduction

Transcatheter aortic valve implantation [TAVI] has signaled a new chapter in the management of valvular heart disease. Resources have mostly been focused on treating senile degenerative disease in mostly affluent populations with rheumatic heart disease [RHD] remaining an orphan disease. However, RHD remains the most common cause of death from valvular heart disease in the world at almost double the rate of non-RHD valve lesions (1).

RHD has been virtually abolished from many developed countries (2) and the preferred approach to management is prevention rather than treating complications (3, 4). However, in countries where RHD has the highest prevalence and mortality, there is inadequate use of proven treatments such as antibiotic prophylaxis (5). Although prophylaxis should be the cornerstone of management, it cannot be ignored that the burden of established valvular disease and complications of RHD are likely to remain with us for decades to come.

Management of patients with significant valvular lesions require surgery that is not freely available in areas where RHD is common and post operative anticoagulation is often poorly administered in such areas (6).

Transcatheter treatments in isolation or combined with surgery hold significant promise as a solution. Most of the current transcatheter treatments are however inappropriate for RHD patients. Rather than waiting for first world solutions to become more applicable, we believe that dedicated solutions should be sought.

This review will evaluate how the anatomy of rheumatic valves differ from other pathologies with a specific view to transcatheter solutions. This will be contextualized against a focused epidemiological discussion of RHD, providing a rationale for considering such interventions. The many shortcomings in current RHD management, interventional management and current transcatheter treatments will be discussed and finally we will look at future developments in the field.

## Scope of the problem

Acute rheumatic fever disproportionately affects children and young adults, with a peak incidence reached between 5 and 15 years of age (7). Recurrent inflammatory damage to heart valves leads to progressive valve dysfunction with established rheumatic valvular involvement peaking between the ages of 20 and 29, and only declining again after the age of 40 (1). The prevalence has not declined much over a 25year period from 1990 to 2015 in many regions where it is most prevalent (>70% of the world's cases occur in sub-Saharan Africa, South Asia, and Oceania) and mortality remains high in South Asia and sub-Saharan Africa (7).

The World Health Organization (WHO) and the Global Burden of Disease study respectively estimate that RHD affects 33 and 40.5 million persons globally, eclipsing the degenerative aortic valve stenosis [AS] burden 4-fold, and with annual death rates exceeding 300,000 cases. Although mortality related to RHD had appeared to decline between 1990 and 2012, a worrying trend has seen mortality rising sharply since 2017 (1, 8).

**Figure 1:**
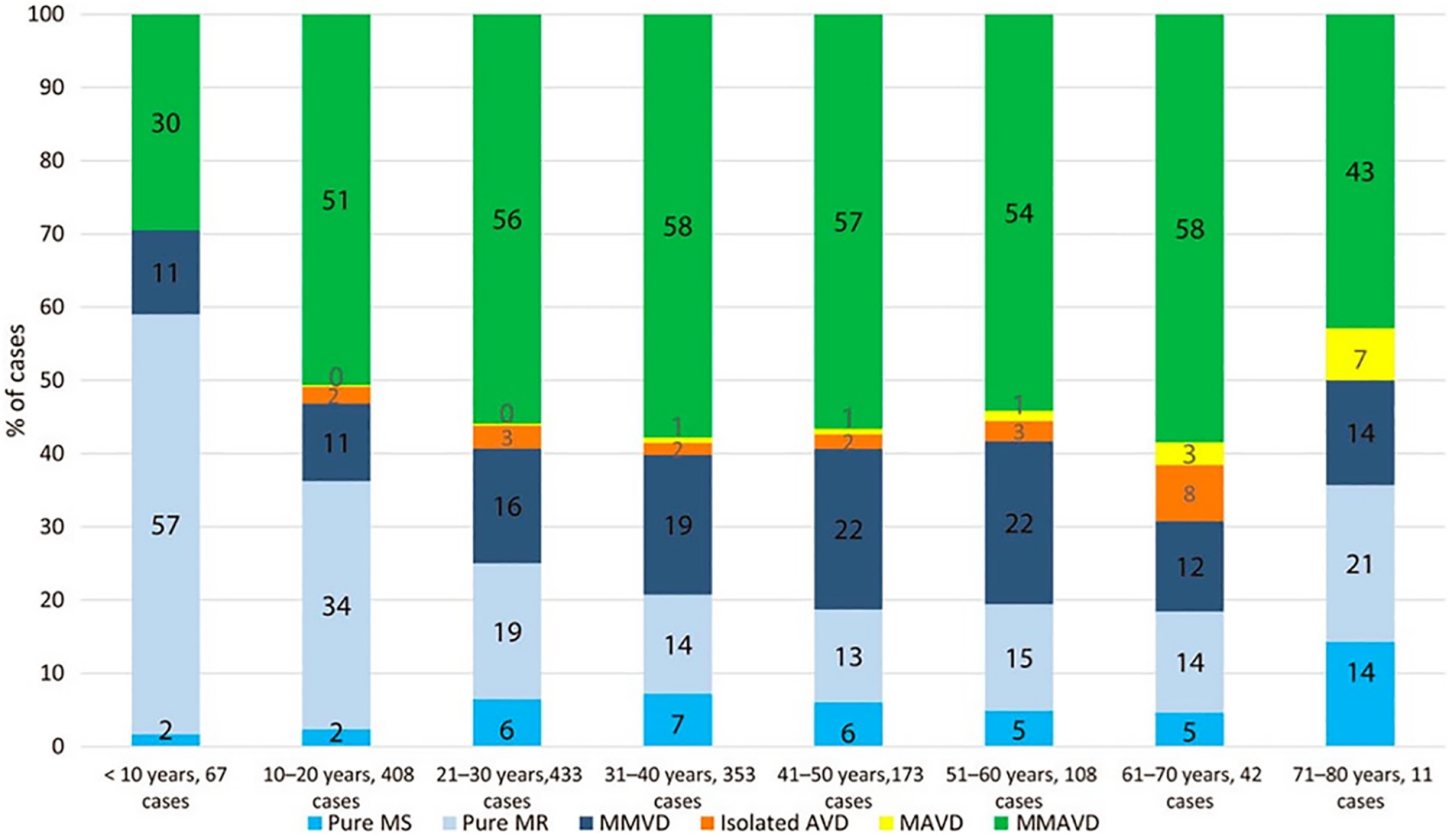
The pattern of native rheumatic valve disease in 2,475 children and adults with no percutaneous or surgical interventions. AVD, aortic valve disease; MAVD, mixed aortic valve disease; MMAVD, mixed mitral and aortic valve disease; MMVD, mixed mitral valve disease; MR, mitral regurgitation; MS, mitral stenosis from (5). Used with permission.

Involvement of the mitral valve is the hallmark of RHD with >95% of cases in epidemiological studies exhibiting mitral valve involvement (9, 10). Chronic RHD of the mitral valve progresses over years and causes mitral regurgitation [MR] early in the disease process and mitral stenosis [MS] later on (11). Aortic valve involvement occurs in 20%–30% of cases, although rarely in isolation, but typically with associated mitral valve involvement (12). The REMEDY study evaluated 3,343 patients with RHD from 14 low- and middle-income countries and found mixed valvular involvement in the majority of cases with the most valve lesions being moderate or severe (5) (Figure 1). Multivalve involvement often leads to difficult management decisions because not all lesions are severe at the time of first surgery and the disease is progressive [will be discussed later].

Epidemiological studies have documented that approximately two thirds of RHD patients are female (13). Stenotic left heart valve lesions are poorly tolerated during pregnancy (14), and it is therefore not surprising that many patients present for the first time with RHD during pregnancy (13). The mortality of untreated severe MS in pregnancy is as high as 34% in countries with limited access to surgery (15) or percutaneous balloon mitral valvuloplasty [PBMV], despite good results with this procedure in pregnancy (16, 17). Management decisions in such woman are often complex and with the high fetal mortality of conventional surgery (18), access to a wider scope of less invasive alternatives [even if used as a temporizing measure] may provide a better outcome.

## The anatomical challenges for transcatheter treatments

The anatomy of rheumatic valves differs significantly from that of degenerative valves which limits the applicability of current transcatheter devices in most RHD populations. The landing zone in the degenerated calcified AS valve provides good anchoring for a TAVI prosthesis and represented relatively low hanging fruit for designers. In the aortic valve, the process of degenerative valve calcification starts with fibrocalcific changes on the aortic aspect of the valves, near the hinge points. From here it progresses through the coalescence of microcalcifications into larger nodules mostly on the aortic leaflet surface (19). In a small study of 39 explanted valves, the calcification of rheumatic valves was more diffuse than in non-rheumatic valves (20). The hallmark of rheumatic AS is fibrotic fusion of the commissures which leads to a potentially different anchor for a transcatheter valve compared to the large nodules of calcium in degenerative valves. In a study of aortic valves, researchers found good correlation between CT scan calcium scores and AS severity in older patients, but not in those <51 yrs of age [where calcification was generally less] (21). This study however did not include any confirmed rheumatic valves and the younger patients generally had bicuspid valves where the pathology is not comparable to RHD in terms of simulating commissural fusion and its progression. This lack of detailed descriptions of calcium distribution in rheumatic valves is a significant obstacle in the development of transcatheter valves for this indication.

The dominant indication for aortic valve replacement in emerging economies remains aortic regurgitation (6, 22, 23) which is ill suited to treatment with current generations of TAVI prostheses. Fibrosis predominates in these valves with less significant calcification to anchor the TAVI prosthesis (24).

The mitral valve structure and function in health is far more complex than that of the aortic valve and diseased mitral valves pose an even bigger obstacle to transcatheter solutions. Morphologic features of rheumatic MS include commissural fusion, thickening of the leaflets and thickening and shortening of the subvalvular apparatus (25). In the earlier stages of the disease, the leaflets are minimally fibrosed in the majority of patients under the age of 30, while in patients over 40 the majority have significant valvular scarring (26). Calcification of the mitral valve in degenerative MS is usually concentrated in the annulus which creates a potential anchor for transcatheter valves (27). In rheumatic MS however, calcification can be limited early in the disease process but accumulates progressively and can be present in any part of the valve, often very asymmetrically (28). Heavy calcification, particularly involving the commissures, decreases the success rate of balloon valvuloplasty and becomes a potential indication for valve replacement (29). From Figure 1 it can be seen that the vast majority of patients present with mixed valve disease which further complicates the evaluation of the anatomy and the design of studies to delineate it with a view to designing transcatheter devices to treat it. See Table 1.

**Table 1:** A summary of the challenges in the application of transcatheter treatments for RHD with potential solutions.

Challenge	Solution
Anatomical challenges	
Lack of data on calcification patterns	Studies utilizing cardiac CT to delineate better
Dominant aortic lesion is AR	Dedicated TAVI anchoring mechanisms
Limited MAC	Dedicated TMVR anchoring mechanisms
Mitral commissural fusion	TMVR that anchors at commissural level
Accurate deployment of balloon expandable TAVI in non-calcified anatomy	NOB to deploy balloon expandable TAVI
Other	
Progressive disease requiring different valve surgeries at different times	Hybrid approach with surgical MVR as first operation and TAVI later
No dedicated TMVR device currently available	Surgical valve dedicated as docking station for transcatheter re-intervention in future
Patients present late with decompensated LV function	NOB to deploy balloon expandable TAVI
Poor durability of bioprosthetic leaflets in young patients	• Improved bioprosthetic tissue treatments • Decellularization • Improved tissue fixation techniques • Polymer leaflets

AR, aortic valve regurgitation; TAVI, transcatheter aortic valve implant; MAC, mitral annular calcification; TMVR, transcatheter mitral valve replacement; NOB, non-occlusive balloon catheter; MVR, mitral valve replacement.

## Established management

Medical management is appropriate for the earlier stages of RHD and the management of concomitant atrial fibrillation [AF]. However, once symptoms and signs of decompensation develop, there is little evidence that medical management alters outcome and exploring interventional treatments become warranted (30). In a large study of low- and middle-income countries, 16,9% of patients died at an average age of 28 within 2 years of being diagnosed. Mortality was significantly higher in lower income countries where access to surgery or percutaneous treatment options were limited (13). This illustrates the scope of the problem and lends important justification for actively exploring alternative treatment strategies.

### Mitral valve disease

The pathology in rheumatic MS is complex and typically represents advanced valvular fibrosis, calcification and commissural fusion. Despite this, commissural fusion without superimposed calcification is very amenable to being cleaved effectively. Initially this was achieved by surgical techniques (31). Subsequently PBMV was established and this is now the preferred option for intervention (25, 32). PBMV has been shown to be at least as effective as the initial surgical techniques but is significantly less invasive (33, 34). For this reason, patients are evaluated for PBMV as an initial strategy and surgery is offered when this is deemed unlikely to succeed (35). The valve morphology is evaluated with echocardiography [and sometimes fluoroscopy to assess calcification] and a variety of scoring systems exist to aid evaluation (36, 37). Medium term results are very good but after 5yrs there is a steady rise in event rates particularly in less suitable valves. In a large study comparing patients with a Wilkins score <8 with those >8, survival (82% vs. 57%) and event-free survival (38% vs. 22%) at 12-year follow-up was better when the score was low (36). A number of clinical and valve related factors that predicted adverse outcomes included a Wilkens echo score >8, age, prior surgical commissurotomy, NYHA functional class IV, pre-BMV mitral regurgitation ≥2+, post-BMV mitral regurgitation ≥3+ and higher post-BMV pulmonary artery pressure (38). The combination of MS and MR is present in 20%–30% of cases (9, 10) and when the MR is moderate, this is an indicator of adverse outcome with PBMV and mitral valve replacement may then be required.

When MR is the predominant lesion, and the patient develops an indication for intervention, surgical repair or valve replacement are currently the only options. Valve repair is only possible in non-calcified valves and requires significant expertise. The results with repair in children and young adults are acceptable (39–41) but is performed in only a small proportion of cases (42). Valve replacement is performed more often but outcomes are limited by the durability of bioprosthetic valves, particularly in younger individuals and complications of anticoagulation when mechanical valves are used. Because of the relatively young RHD population, mechanical valves are favored [65%–85% of cases in adult populations] (43, 44) and when compared to repair cohorts, patients who receive mechanical valves tend to be older patients and have more mixed valvular pathology and AF (45). Heart valves in the mitral position are less durable because they are subjected to different hemodynamic loads than the aortic position. While mitral bioprostheses degenerate faster because of greater loading forces (46), mechanical prostheses are two to three times more likely to thrombose (47).

The RHD population often include patients from poorly serviced areas and problems with anticoagulation have led some authors to recommend the use of tissue valves in certain populations, particularly young females who have not completed their families (48). Such patients are then very likely to require redo surgery, which is more complex and carries up to double the mortality of first operations (49). Transcatheter valve-in-valve procedures may have a role in these patients.

### Aortic valve disease

Although true isolated aortic valve disease is rare in RHD, approximately one third of RHD patients have aortic valve involvement in combination with mitral valve disease (13, 50). In these patients, AR is much more common than AS. Most patients however require mitral valve surgery in isolation as a first operation (51) and the place for transcatheter aortic valve interventions may lie with the group of patients who present with aortic valve disease after an initial mitral valve intervention. One would unfortunately have to speculate on the true requirement for this as current literature simply does not provide answers. In one of the very few studies that provides some insight, Russell et al. looked at a large Australian database of 17,000 heart valve surgeries, including 1,384 cases of RHD. Compared to non-RHD patients, it was found that patients undergoing RHD surgery were significantly younger and more likely to be female, of indigenous ethnicity and have had prior PMBV or surgery. Indigenous Australians were 15 times more likely to come from remote areas [with problematic anti-coagulation], partly explaining why they were more likely to receive valve repairs or bioprosthetic valves. Although 16% of all RHD operations were redo operations, we do not know how many were redo operations for a different valve. Twenty three percent of patients underwent isolated aortic valve operations and 20% combined aortic and mitral valve operations (51). One might therefore speculate that there is likely to be a moderate sized group of patients that may benefit from transcatheter aortic valve interventions after previously receiving either PMBV or mitral valve replacement.

## Challenges of current interventional management

Once valve lesions become severe and symptoms develop, intervention is generally indicated but even this well-established approach is hampered by a number of factors in the RHD population.

**Figure 2:**
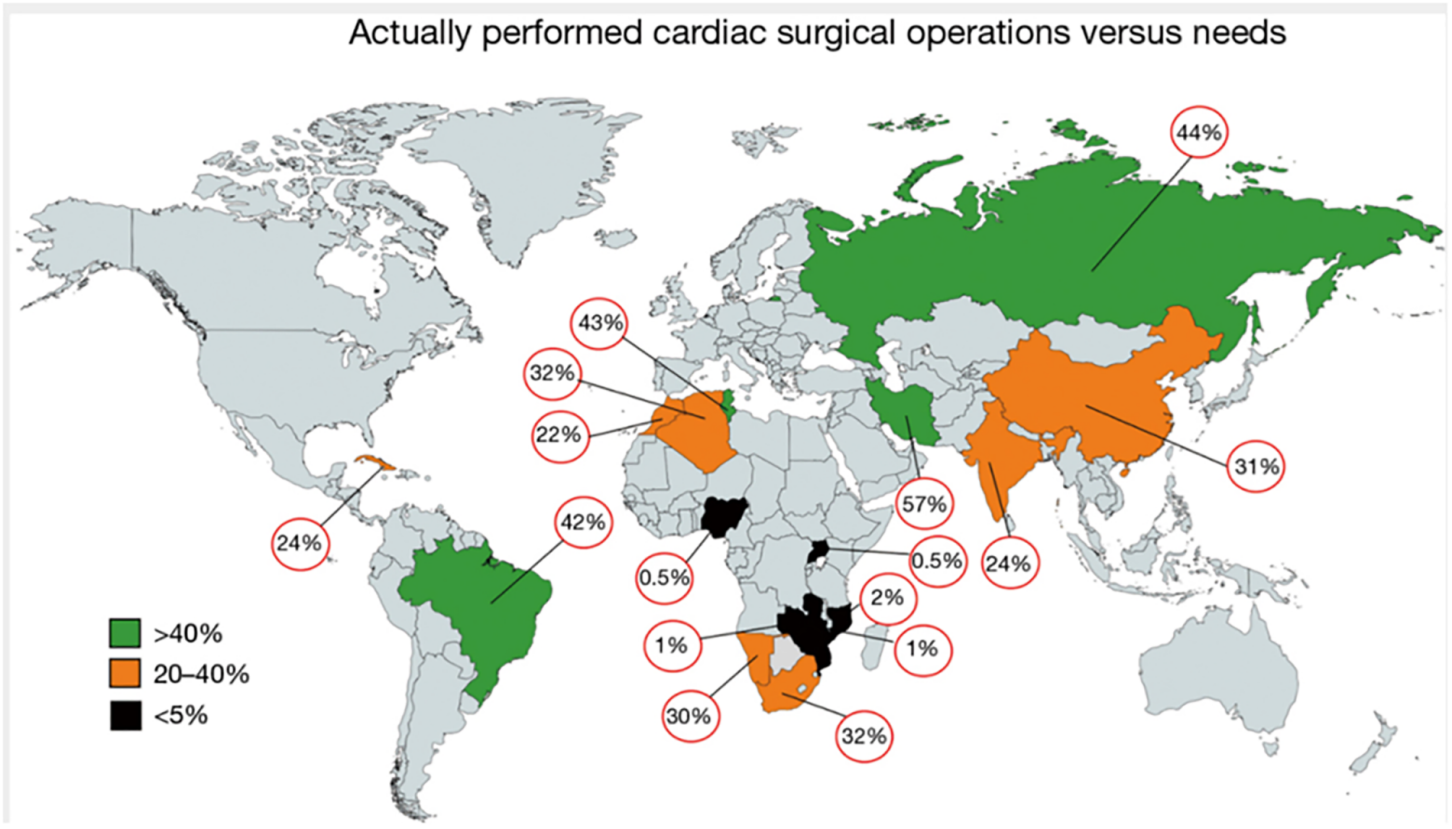
Level of actually performed cardiac surgery as a percentage of the needs for surgery in the individual country. This data was generated from a study investigating the current state of cardiac surgery in a variety of low- and middle-income countries. It clearly illustrates the dire shortage of access to surgery in Sub-Saharan Africa. The percentages depicted represent mean values thereby masking country-specific social, geographic or political diversities. From (22). Used with permission.

One of the biggest challenges in the management of RHD is the limited access to open-heart surgery in the areas where RHD is still rife. The vast majority of new, less invasive developments are aimed at treating patients from affluent countries where 85% of the world's open-heart surgeries are performed on 11% of the world's population (52). A number of studies have now shown an alarming lack of access to surgery in the countries with the highest burden of disease requiring such surgeries. In Sub-Saharan Africa, there is one cardiac surgeon per 14.3million population compared to one per 1.1 million in North Africa and one per 0.1 million in Brazil (53). The number of surgical procedures is similarly lacking in Sub-Saharan Africa (6, 53). See Figure 2. In another study, only 11% of patients from low-income countries received a valve repair or replacement, compared to 60,8% of patients in upper-middle-income countries. Approximately 90% of patients received mechanical valves and although most were prescribed oral anti-coagulants, 12% had no INR monitoring and 34% had three or less INR measurements in the preceding 6 months (13). Apart from poor monitoring, the complication rates of these valves are high and even in an upper-middle income country, up to 1 in 4 young patients with RHD receiving a mechanical AVR have a major valve-related event within the first decade after the procedure (6, 53). Ineffective anti-coagulation has been associated with poverty, low levels of education and larger distances to monitoring clinics (54). This problem is likely to remain as only +/- 10% of Western patients are still receiving mechanical valves (55), which explains why the most commonly used mechanical valve has remained largely unchanged for more than 40 years (56). Improvements in anti-coagulation have included home INR monitoring which is not available to most patients in low-income countries, and non-vitamin K oral anticoagulants [NOACs] which has disappointingly been found to be suboptimal for these patients and are therefore not recommended by major guidelines for use in valve prostheses (35, 57). These inadequacies in access to surgical management raise the question of whether access to catheter-based interventions will be any better. There are however countries in sub-Saharan Africa [such as Zimbabwe, Zambia, and the Democratic Republic of the Congo] where interventional cardiology services have recently been established for the first time. This growth in catheterization laboratories may hold the answer, provided that procedures and devices can be developed that are applicable for RHD patients.

PBMV is probably the most cost effective and least invasive intervention for RHD valve pathology and should be considered a priority to provide. Unfortunately, the opposite is true with only 1.1% of RHD cases in low-income countries receiving it, compared to 8% in upper middle income [African] countries. One reason for this is that many patients do not have access to a catheterization laboratory. Unfortunately, patients present late in the disease course and PBMV is often not suitable, as evidenced by the 7-fold higher rates of surgery [compared to PBMV] reported in the REMEDY study (5, 13).

Another significant problem is that RHD is a progressive disease, and one is often confronted with a patient who has had one valve replaced previously and now presents with significant dysfunction of another valve. This requires high risk re-do surgery and often very difficult decision making. The alternative approach of operating on mild aortic valve disease at the time of the mitral valve replacement has been found to yield no benefit and the current recommendation is to leave other mild valve disease at the time of the initial operation (58). Re-do mitral valve surgery in one South African study was performed a mean of only 4 years after the initial surgery and in 73% of these cases the surgery was performed as an emergency. The majority of these [80%] were due to valve thrombosis, in fact, 25 of the 26 patients with multiple redo surgeries had valve thrombosis with reportedly high rates of post-operative complications (59).

## Challenges of current transcatheter therapies

### Aortic valve

Transcatheter balloon aortic valvuloplasty for rheumatic AS is performed relatively infrequently with significantly less case selection guidance from literature than for MS. When the anatomy is deemed suitable, the results are however acceptable with 85% of patients obtaining a >50% reduction in gradients of the valve in one large study (60). These results were sustained at 5 years and only 2% developed severe regurgitation.

Although the use of transcatheter valves for AS due to RHD has been described in the literature, it is limited to case reports and small series (61, 62). In the largest cohort described to date >1,000 TAVI patients with rheumatic aortic valve involvement were obtained from the Medicare database (63). The diagnosis of RHD in this study was dependent on correct ICD-10 codes and therefore is open to criticism. Although the results did not differ significantly between rheumatic and non-rheumatic valves, the patients in both groups were elderly [79 and 81 years old] and the behavior of a TAVI prostheses at this stage in the disease course is likely to be similar. The applicability of these results to younger populations is therefore questionable. Unfortunately, this study did not report on other valve involvement which would have added to the diagnostic accuracy of RHD and could provide information on the real-world dilemma of multiple valve involvement and previous valve surgery in TAVI candidates. The lack of anatomical and clinical data in the RHD population raises a number of questions when we consider the applicability of current TAVI prostheses to the RHD population.

Anchoring of the TAVI prosthesis in the heavily calcified landing zone in senile degenerative AS is now well established. It is however not known whether the performance of the valves will be the same in a rheumatic landing zone with commissural fusion and potentially different patterns of calcification. Published case series included older patients with mean ages between 79 and 83, when calcification is likely extensive (61, 63, 64) and the dominant lesion is AS. The predominant aortic valve lesion in low- and middle-income countries is however AR (6) and most current prostheses are either not approved or have shown inferior results in AR patients as compared to AS. In a systematic review by Yousef et al, a total of 175 cases of TAVI implantation for AR from 31 studies were included and demonstrated valve malposition [3,4%], second valve required [11.3%], residual AR grade ≥2+ [17.7%] and conversion to SAVR [2.3%] (65). These rates do not compare well with contemporary practice in AS patients (66, 67). The only valve currently approved for use in isolated aortic regurgitation [AR], is the JenaValve [Irvine, CA] (68) which has anchoring arms that grip the native aortic valve leaflets. It may therefore be applicable to the anatomy of some RHD patients but more research exploring novel approaches to anchoring TAVI valves is required.

The durability of current bioprosthetic valves [aortic and mitral] in younger patients is unacceptable for most clinical scenarios (69). Numerous approaches to overcome this problem are being investigated: new tissue fixation techniques have been tested in animal models (70–75) but translating these very early developments into a functional valve approved for human implantation is time consuming and has not realized.

The cost of all current TAVI prostheses is prohibitive in most settings where RHD is prevalent. Studies comparing the cost of SAVR with TAVI have found comparable total hospitalization cost in first world elderly populations (76). The higher TAVI prosthesis expense is offset by shorter hospital stay and is used, at least in part, as justification for the extremely high cost of all current TAVI systems. This argument does not hold for younger, lower risk RHD patients where one would expect lower total hospitalization costs for surgery (70, 77). Although there are no robust cost analyses for the cost of cardiac surgery in Africa compared to the first world, there is some data to indicate that it could be performed at a relatively affordable cost (78) and the price of TAVI devices aimed at RHD patients would therefore have to be more affordable.

Transcatheter heart valves require large bore arterial access [typically 18Fr]. The management of this access requires skill and potentially expensive bailout equipment such as covered stents, balloons, snares, and vascular closure devices. This is not widely available in low-income settings.

### Mitral valve

Since the advent of PBMV, a large body of evidence in support of it has accumulated. Although the results are very good in appropriate candidates, access to the procedure and expertise is limited—as discussed above. Attempts at improving on the original Inoue technique (32) have included double balloon (79), using lithotripsy to fracture calcification (80), over-the-wire techniques (81) and the use of intracardiac echocardiography to guide the procedure (82). The Inoue technique remains the most used and because it works so well, is unlikely to change too drastically. The shortcoming of this technique is more one of logistics: improved access to it and detecting disease earlier when PMBV is still possible.

**Figure 3:**
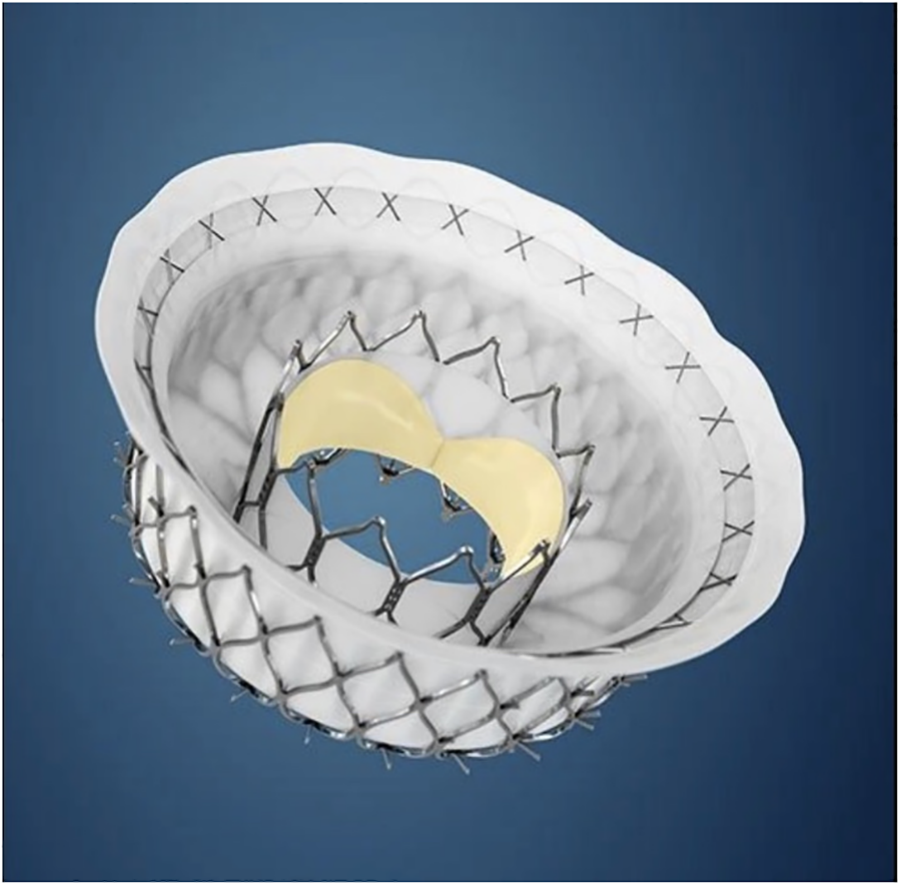
The intrepid transcatheter mitral valve [Medtronic, MN]. Note the double stent design with the outer flexible anchor stent [with small hooks on outside] which isolates the inner valved stent from ventricular compression and therefore may improve durability. Image supplied by Medtronic.

**Figure 4:**
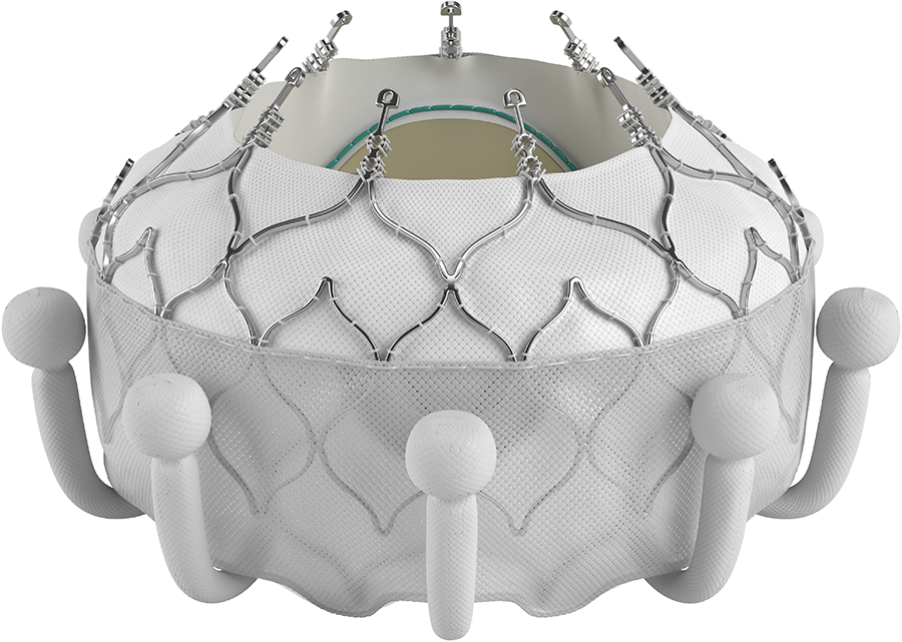
The EVOQUE transcatheter mitral valve [Edwards Lifesciences LLC, Irvine, CA]. Note the anchors that engage the leaflets and subvalvular anatomy to secure placement. This system is designed to treat non-rheumatic mitral valve regurgitation and is not indicated for rheumatic heart disease. Image supplied by Edwards Lifesciences.

Other transcatheter treatments techniques to intervene on non-rheumatic mitral valves [such as transcatheter edge-to-edge repair and transcatheter annuloplasty] is not applicable to RHD because of anatomical limitations (83) and cost. The solution would therefore more likely be the development of applicable transcatheter mitral valve replacement [TMVR] devices. No current TMVR design is intended for treatment of rheumatic mitral valve disease and all the valves with human implant data are primarily intended for degenerative mitral regurgitation (84–87). Despite this, the impressive engineering that has gone into their development should offer important lessons that may aid in the design of dedicated RHD mitral valve devices. One such feature is the double stent design where an outer, flexible stent anchors the valve in the annulus and isolates the inner valved stent from systolic compression by the ventricle. See Figure 3. Another important design feature is different anchoring mechanisms such as either sharp hooks that penetrates into the annulus or a nitinol self-expanding frame with ventricular anchors that engage the leaflets and subvalvular anatomies to secure placement of the EVOQUE valve [Edwards Lifesciences LLC, Irvine, CA] -see Figure 4. How this type of design will interact with a diseased subvalvular apparatus remains unclear. Although these designs are a step in the right direction, their general applicability to the RHD population is doubtful for a number of reasons. Annular calcification that might aid anchoring of a transcatheter valve is less prevalent. In fact, significant annular calcification is uncommon. In a pathology study, only 23% of excised rheumatic mitral valves had significant annular calcification. If the dominant lesion was however MS, 80% had significant calcification (28). Studies evaluating calcification on CT-scans include very few RHD cases but indicated that some calcification is visible in the leaflets of MS patients and with older age and more severe MS, the presence of annular calcification increases (88).

Commissural fusion is the hallmark of rheumatic MS and is likely to be a prominent factor in most candidates for TMVR. Because of the complex three-dimensional shape of the mitral valve, commissural fusion is likely to grip the prosthesis at the level of the native leaflet tips, rather than the annulus and designs may have to incorporate this to ensure secure anchoring. Although implantation of a TAVI device has been described in a rheumatic mitral valve with very little annular calcification, this was in an 80 yr old patient and therefore represents a unique case with limited generalizability (89).

One of the major drivers of mortality post TMVR is LVOT obstruction which is partially due to systolic anterior motion of the anterior mitral valve leaflet (90). Attempts to predict this complication may have improved outcomes but it remains a major limitation in the development of this field (90). It is not known how significant rheumatic subvalvular apparatus involvement will influence this complication but theoretically, it may be protective if the anterior mitral valve leaflet is retracted and immobile and therefore pulled out of the LVOT.

## Future developments

There appears to be a large potential for transcatheter developments directed at patients with RHD.

Although the list of challenges is extensive, a few teams have made some progress to address two of them: deliverability/anchoring and durability. What is encouraging is that many of these developments originate in countries where RHD is prevalent (72, 73, 75, 91–96).

**Figure 5:**
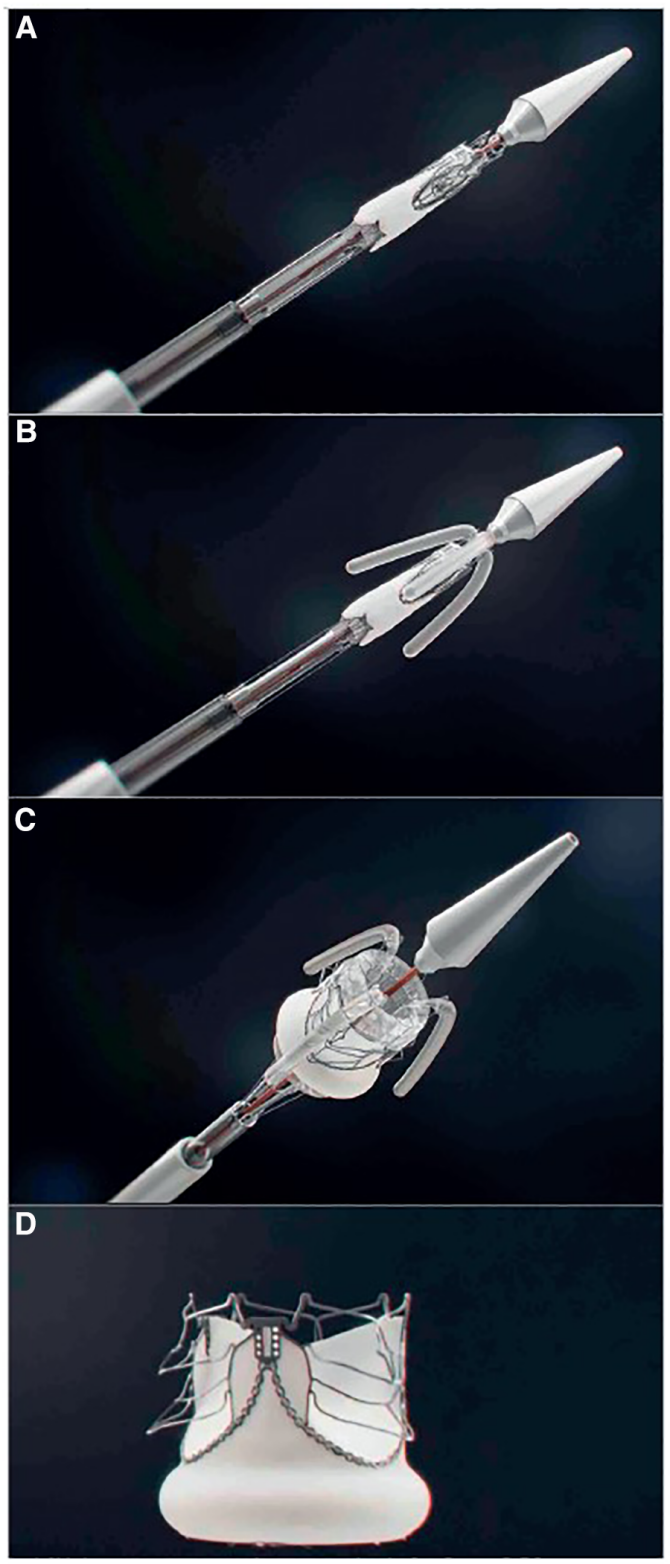
Key stages of the deployment of the self-homing, nonocclusive SAT-TAVI valve. Crimped SAT-TAVI system pushed out of the deployment sheath (**A**), with the locator and stabilizer trunks deployed (**B**) followed by the full expansion of the scalloped, self-anchoring stent (**C**) The cobalt-chromium stent is designed to lift up six arms through plastic deformation (**D**) All arms are seated supra-annularly creating sinus-like outward bulges of the leaflets that firmly anchor the stent in the absence of leaflet calcification. From (91). Used with permission.

**Figure 6:**
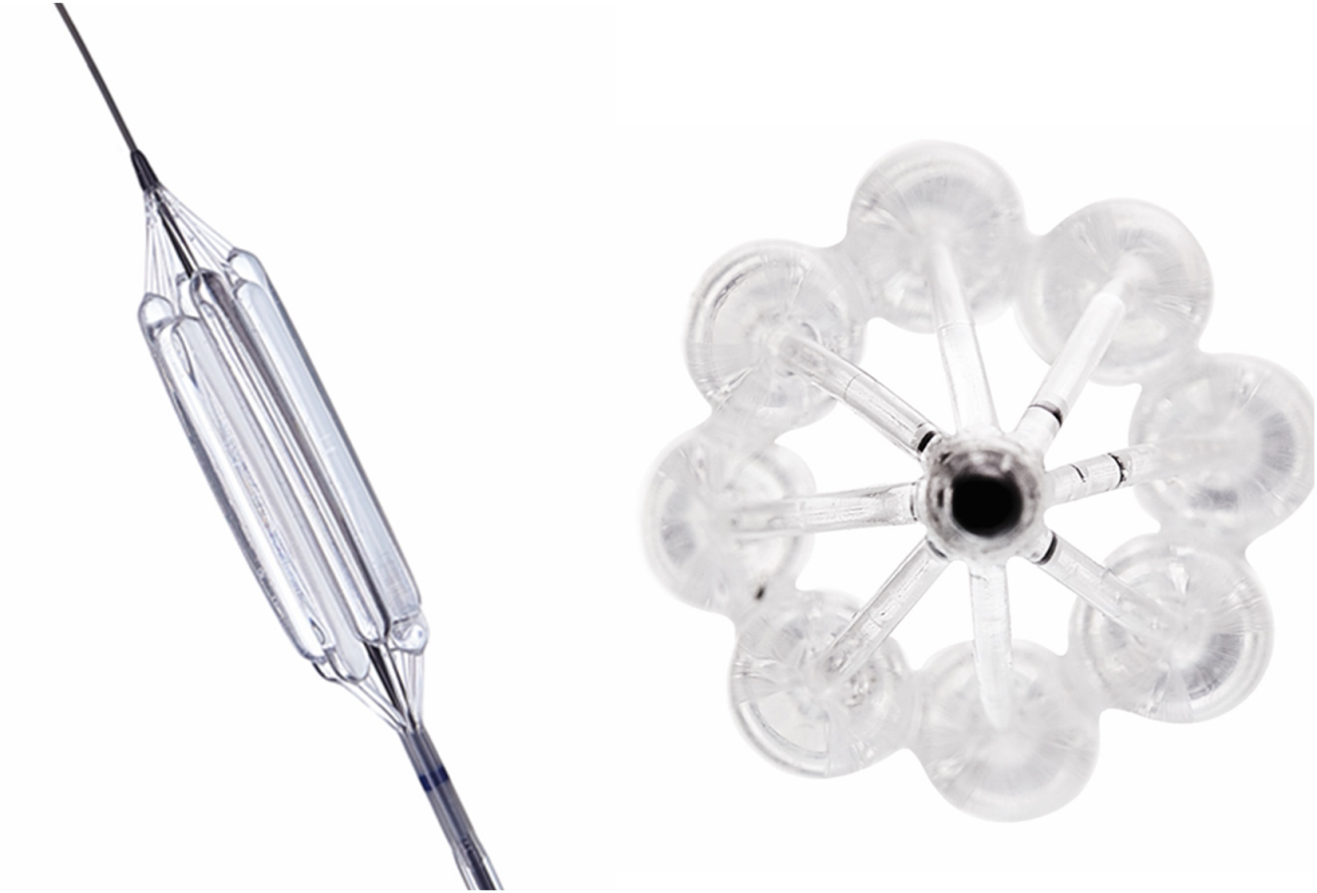
The outflow non-occlusive balloon catheter [DISA Medinotec, South Africa]. On the left is a photograph of the device from the side and on the right is an end on view showing 8 smaller balloons arranged in a circular fashion to allow blood flow down the central channel during expansion. This may be more stable during valve deployment without the need for rapid ventricular pacing (96). Pictures supplied by DISA Medinotec.

If we consider that since the greatest need is for mitral valve prostheses and that we are probably furthest away from a viable transcatheter valve for this indication, a hybrid approach may be the first step. A surgically implanted bioprostheses designed specifically as a docking station for future transcatheter re-intervention should be investigated. Implanting a transcatheter valve inside a degenerated bioprosthetic valve is less challenging because the landing zone is radio-dense, and anchorage should be simpler. Furthermore, the native mitral valve apparatus is less likely to interfere with the LVOT. This surgical valve would however still require improvements in durability. The transcatheter alternative to this hybrid approach is unfortunately a field with the least progress and to our knowledge, there are no published reports of transcatheter mitral valves designed specifically for RHD. This is hardly surprising, given the complexity of the mitral anatomy and initial attempts have therefore focused on the aortic valve. The team of Zilla et al. in Cape Town has the only reported TAVI device with animal implant data designed specifically for RHD. This device addresses the issue of deliverability and anchoring with several unique features [see Figure 5]: to enable positioning in a non-calcified landing zone and align the cusps with those of the native aortic valve, three locator trunks are first deployed, and the valve is then inflated with a lumen-preserving, non-occlusive balloon that does not require rapid pacing. During expansion, three sets of arms protrude from the valve to anchor it in the sinuses in the absence of calcification. Although this device requires transapical access and is still in the preclinical phase, it represents a significant first step in the right direction (91). The utility of a non-occlusive balloon is based on the belief that many of the patients with RHD present at the stage of inoperability because of ventricular decompensation. A balloon expandable valve that does not require rapid ventricular pacing may therefore be of benefit (92). It is not known whether this will eventually be a significant benefit, but similar balloons have been tested (96) and may be utilized in pre- or post-dilatation of valves. See Figure 6.

The need for anti-coagulation with all its problems and the need to crimp valves, disqualify mechanical valves from utility in the field of transcatheter heart valves. The durability problems with flexible leaflets have been the subject of a lot of research, which has focused on improving pericardial tissue and non-biological alternatives. Pericardium harvested from animals needs to be fixed with glutaraldehyde [GA] which provides mechanical stability to the tissues and reduces antigenicity, but at the cost of increasing susceptibility to calcification and impairment of growth potential (97). Cellular toxicity is associated with the free aldehyde groups of GA, which contributes to preventing the repopulation of tissue with host cells (98). De-toxification of GA fixed pericardium through binding these aldehyde groups have been shown to reduce calcification of tissue (99). Another area of research is decellularization of tissue which involves the removal of host cells and nuclear material while keeping the extracellular matrix intact and thereby reducing antigenicity and potentially improving durability (72, 75, 100).These efforts are valuable in our search for alternative bioprosthetic materials but the development/testing process that starts with subcutaneous rat implants and progresses through arterial patches to valves in animals and fatigue testers and then to elderly humans and eventually the wider population. This progresses over decades and then requires outcome studies requiring more decades to fully assess its utility.

Although the first synthetic flexible leaflet heart valve was implanted as far back as 1960 (101) and despite countless efforts to improve on the poor early results (102), development in this field has been fraught with problems such as mechanical deterioration and calcification of leaflets. The potential for a valve with a long shelf life, no anticoagulation requirement and excellent durability keeps research in the field active. There are many valves in the development phase but only the siloxane-based polyurethane-urea TRIA LifePolymer (Foldax USA) surgical aortic valve has human implant data with 15 implants and good outcomes at 1year (103). This group have also developed a TAVI prosthesis with some animal data based on the same LifePolymer (104). To expand polymer leaflet technology to transcatheter valves requires additionally that the leaflets be thinner and resistant to the crimping process requirement—ideally for prolonged periods to simplify pre-procedural preparation. Recent reports indicate that crimping has an influence on the structure of bioprosthetic tissue (105) but there is only one study with extensive data on the effect of crimping on a polymeric valve [the PolyNova TAVI valve] where the leaflet structure remained stable despite being in the crimped state for up to 8 days (106). A number of polymer valves have shown durability *in vitro* fatigue testers for longer than the required 200 million cycles [as per ISO 5840 requirements] (107) and have also undergone animal implants (91). These valves probably hold the most likely solution to the RHD transcatheter treatment conundrum although history have taught us that we remain further away from an answer than our optimism would want us to believe.

## Conclusion

RHD and its consequences represent a very large clinical and social burden which are likely to be with us for a long time to come. Current treatment modalities fall far short in a number of areas. Major stumbling blocks in the development and implementation of transcatheter solutions for RHD include:

• a paucity of data on the unique anatomy of rheumatic valves
• the high cost of developing new prostheses.
• a perceived lack of financial gain in finding solutions.
• the lack of infrastructure and skills to implant these valves/devices.
• anchoring transcatheter prostheses in a non-calcified environment.
• a critical problem to solve will be the development of foldable valve leaflets that have sufficient durability to be utilized in young patients. Improvements in pericardial tissue fixation is likely the first step but the most likely answer may be the development of synthetic leaflet materials.
